# A computational walk to the hidden peaks of protein performance

**DOI:** 10.1093/synbio/ysab011

**Published:** 2021-05-14

**Authors:** Sonja Billerbeck

**Affiliations:** Molecular Microbiology, Groningen Biomolecular Sciences and Biotechnology Institute, University of Groningen, Groningen, The Netherlands

Spiders use them to catch their prey, plants rely on them to fix carbon and mammals need them for eye vision—proteins.

Proteins play critical roles in nature, and not surprisingly, synthetic biologists heavily rely on their functional diversity to build new therapeutics (1), catalysts (2) and materials (3). But natural proteins are rarely optimal for their envisioned human uses. They rather need to be engineered to enhance their performance. Recently, researchers introduced a machine-learning guided paradigm that can predict which mutations in a protein will enhance function with only 24 functional data sets as input (4). This paradigm could significantly accelerate the engineering of improved proteins for medicine, food, agriculture and industrial applications.

The desire to optimize a protein’s function has always been a centerpiece of synthetic biology, and for decades, protein engineers have innovated the capacities of directed evolution (2) and rational protein engineering. One prominent bottleneck for the engineering of proteins is the difficulty in understanding a protein’s so-called fitness landscape. That means to know, which mutation will make a protein better, while in fact, most mutations render a protein dysfunctional.

The function of a protein is dictated by its amino acid sequence, and protein scientists picture the relationship between sequence and function of a protein as if it was a rugged landscape with shallow hills and high peaks, separated by valleys (5). Valleys represent sequence variants that are not functional, while the highest peaks represent the most functional mutations. Protein engineers now seek to walk through this landscape—each step being one mutation away from the wild-type sequence—in order to explore if they can find higher peaks of performance in sequence space. As the shape of the landscape is mostly unknown, the walk is random and requires the generation of many sequences and the evaluation of their function. Generating this data is often experimentally difficult or expensive. Most importantly, very distant regions of the landscape, where functional peak performance might hide, are not accessible by this search.

Recently, researchers have started to perform this walk through a protein’s sequence space computationally, using deep learning (6). Although several success stories have been reported, each case still relies on a large number of experimental input data. The Church group at Harvard Medical School and the Wyss Institute for Biologically Inspired Engineering now developed a way to mitigate the notorious shortage in experimental data that constrains the engineering of many proteins, by making use of the vast number of publicly available protein sequence data (4, 7).

Instead of learning the fitness landscape of an individual protein from experimental data, they first built a deep learning algorithm that extracts the fundamental features of all functional proteins from the >20 million available unlabeled amino-acid sequences in the UniREF database (7). As such, the algorithm learns what a functional protein sequence likely looks like, enabling exclusion of vast dysfunctional sequences from the search. The search is then fine-tuned when the algorithm learns features specific to the protein of interest, via sequence data from homologues proteins.

Eventually—after having learned from all the available data—the algorithm only requires very few experimental data points to learn a good representation of the protein of interest’s sequence-function landscape: 24 or 96 functionally characterized mutants. The combined algorithm then performs *in* *silico*–directed evolution and suggests protein sequences with various user-defined mutational loads that are likely better performing than the wild-type.

The researchers showed with two evolutionary and functionally different model proteins—avGFP (eukaryotic fluorescent protein) and TEM-1 ß-lactamase (prokaryotic enzyme)—that 5–65% (avGFP) and 2.5–26% (ß-lactamase) of the suggested designs performed better, some up to 10-fold. The hit rate and fold difference in performance thereby depended on the chosen mutational load: the more mutations allowed, the lower the hit rate but the higher the potential gain in performance. Most importantly, those designs included regions in sequence space that had not been accessed by experimental exploration.

In summary, the results suggest the feasibility of what the authors call a ‘24-to-24 design’: in order to get one to two protein variants (95% confidence) that perform better than the original protein, a researcher would just need to generate 24 characterized training mutants and synthesize and characterize 24 suggested designs. Given the ever-decreasing price for gene synthesis, this low number of required input data makes it likely that the paradigm can be applied to many proteins of interest, including those which are currently inaccessible for evolution-based protein engineering as no high-throughput assays are available.

The resource opens up even further opportunities: the algorithm was tested with improving the original function of a protein. But can it be used to facilitate the engineering of new protein functions, for example, as recently experimentally achieved for the direct enzymatic catalysis of a C1–C1 condensation? (8) Also, exciting fundamental questions could be explored: is it possible to improve any protein for human uses? The example of the carbon fixing enzyme RuBisCO suggests that in some cases nature might have already found functional peak performance (9, 10).
